# DC-SIGN targets amphotericin B-loaded liposomes to diverse pathogenic fungi

**DOI:** 10.1186/s40694-021-00126-3

**Published:** 2021-12-24

**Authors:** Suresh Ambati, Tuyetnhu Pham, Zachary A. Lewis, Xiaorong Lin, Richard B. Meagher

**Affiliations:** 1 Department of Genetics, University of Georgia, Athens, GA 30602 USA; 2 Department of Microbiology, University of Georgia, Athens, GA 30602 USA

**Keywords:** C-type lectins, *CD209*, Dendritic Cell-Specific ICAM-3-Grabbing Non-Integrin 1, Targeted antimicrobial drugs, Liposomes, Pathogenic fungi, *DectiSomes*

## Abstract

**Background:**

Life-threatening invasive fungal infections are treated with antifungal drugs such as Amphotericin B (AmB) loaded liposomes. Our goal herein was to show that targeting liposomal AmB to fungal cells with the C-type lectin pathogen recognition receptor DC-SIGN improves antifungal activity. DC-SIGN binds variously crosslinked mannose-rich and fucosylated glycans and lipomannans that are expressed by helminth, protist, fungal, bacterial and viral pathogens including three of the most life-threatening fungi, *Aspergillus fumigatus*, *Candida albicans and Cryptococcus neoformans.* Ligand recognition by human DC-SIGN is provided by a carbohydrate recognition domain (CRD) linked to the membrane transit and signaling sequences. Different combinations of the eight neck repeats (NR1 to NR8) expressed in different protein isoforms may alter the orientation of the CRD to enhance its binding to different glycans.

**Results:**

We prepared two recombinant isoforms combining the CRD with NR1 and NR2 in isoform DCS12 and with NR7 and NR8 in isoform DCS78 and coupled them to a lipid carrier. These constructs were inserted into the membrane of pegylated AmB loaded liposomes AmB-LLs to produce DCS12-AmB-LLs and DCS78-AmB-LLs. Relative to AmB-LLs and Bovine Serum Albumin coated BSA-AmB-LLs, DCS12-AmB-LLs and DCS78-AmB-LLs bound more efficiently to the exopolysaccharide matrices produced by *A. fumigatus, C. albicans* and *C. neoformans* in vitro, with DCS12-AmB-LLs performing better than DCS78-AmB-LLs. DCS12-AmB-LLs inhibited and/or killed all three species in vitro significantly better than AmB-LLs or BSA-AmB-LLs. In mouse models of invasive candidiasis and pulmonary aspergillosis, one low dose of DCS12-AmB-LLs significantly reduced the fungal burden in the kidneys and lungs, respectively, several-fold relative to AmB-LLs.

**Conclusions:**

DC-SIGN’s CRD specifically targeted antifungal liposomes to three highly evolutionarily diverse pathogenic fungi and enhanced the antifungal efficacy of liposomal AmB both in vitro and in vivo. Targeting significantly reduced the effective dose of antifungal drug, which may reduce drug toxicity, be effective in overcoming dose dependent drug resistance, and more effectively kill persister cells. In addition to fungi, DC-SIGN targeting of liposomal packaged anti-infectives have the potential to alter treatment paradigms for a wide variety of pathogens from different kingdoms including protozoans, helminths, bacteria, and viruses which express its cognate ligands.

**Supplementary Information:**

The online version contains supplementary material available at 10.1186/s40694-021-00126-3.

## Background

DectiSomes are lipid nanoparticles carrying an anti-infective and coated with a protein that targets them to a pathogen [1, 2]. The goal in the design of DectiSomes is to increase the local drug concentration at the pathogen’s surface and reduce the drug concentration delivered to host cells [1]. We’ve previously shown that two classes of DectiSomes, Amphotericin B (AmB) Loaded Liposomes (AmB-LLs) coated with either of two C-type lectin pathogen receptors Dectin-1 or Dectin-2 are concentrated on fungal pathogens and have improved antifungal efficacy. In particular, the carbohydrate recognition domains (CRDs) of these two proteins bind fungal oligoglucans and oligomannans, respectively. Dectin targeted AmB and Anidulafungin loaded liposomes (e.g., DEC1-AmB-LLs and DEC2-AmB-LLs) are order(s) of magnitude more effective than AmB-LLs at binding and killing *Aspergillus fumigatus, Candida albicans,* and/or *Cryptococcus neoformans* in vitro [2–4]. DEC2-AmB-LLs are dramatically more effectively at controlling infections in mouse models of pulmonary aspergillosis [1, 5] and invasive candidiasis [2] than untargeted AmB-LLs. The Dectins recognize most fungal pathogens [6] and many non-fungal pathogens [7]. We wished to expand our exploration to other potential targeting proteins that recognized fungal pathogens and an even wider variety of non-fungal pathogens.

Human DC-SIGN (a.k.a., CD-SIGN, ICAM-3*)* is C-type lectin pathogen receptor encoded by the *CD209* gene*.* Its CRD binds variously crosslinked mannose-rich and fucosylated glycans (e.g., the Lewis^X^ trisaccharide), and lipomannans often found in protein conjugates [8–11]. Consequently, DC-SIGN’s CRD recognizes cognate glycans in diverse fungal [6], viral [12], helminth [13], protist [14], and bacterial [15] pathogens, and thus has the potential for liberal pathogen targeting of liposomes. DC-SIGN is expressed by Antigen-Presenting Cells and a few other cell types. In dendritic cells, when DC-SIGN’s extracellular CRD binds to its cognate ligands, its intracellular domain signals both innate and adaptive immune responses to infection [6, 16, 17]. Because mouse genomes encode eight DC-SIGN homologs, but no clearly defined ortholog of human DC-SIGN [18], we initiated this project with human DC-SIGN, even though we will test it here in mouse disease models.

Among various fungal pathogens recognized by DC-SIGN [6], *Aspergillus spp., Candida spp.,* and *Cryptococcus spp.,* cause a majority of global life-threatening invasive fungal infections and hundreds of thousands of deaths annually [19, 20]. These three genera partially represent the extreme diversity in the fungal kingdom, because they diverged from common ancestors approximately 0.8 to 1.3 billion years ago [21]. These fungi are responsible for approximately 4.5 billion dollars in U.S. medical costs annually [22], further supporting their importance as models for our study. Herein, we examine DC-SIGN’s targeting of liposomes to representatives of these three fungal genera.

**Fig. 1:**
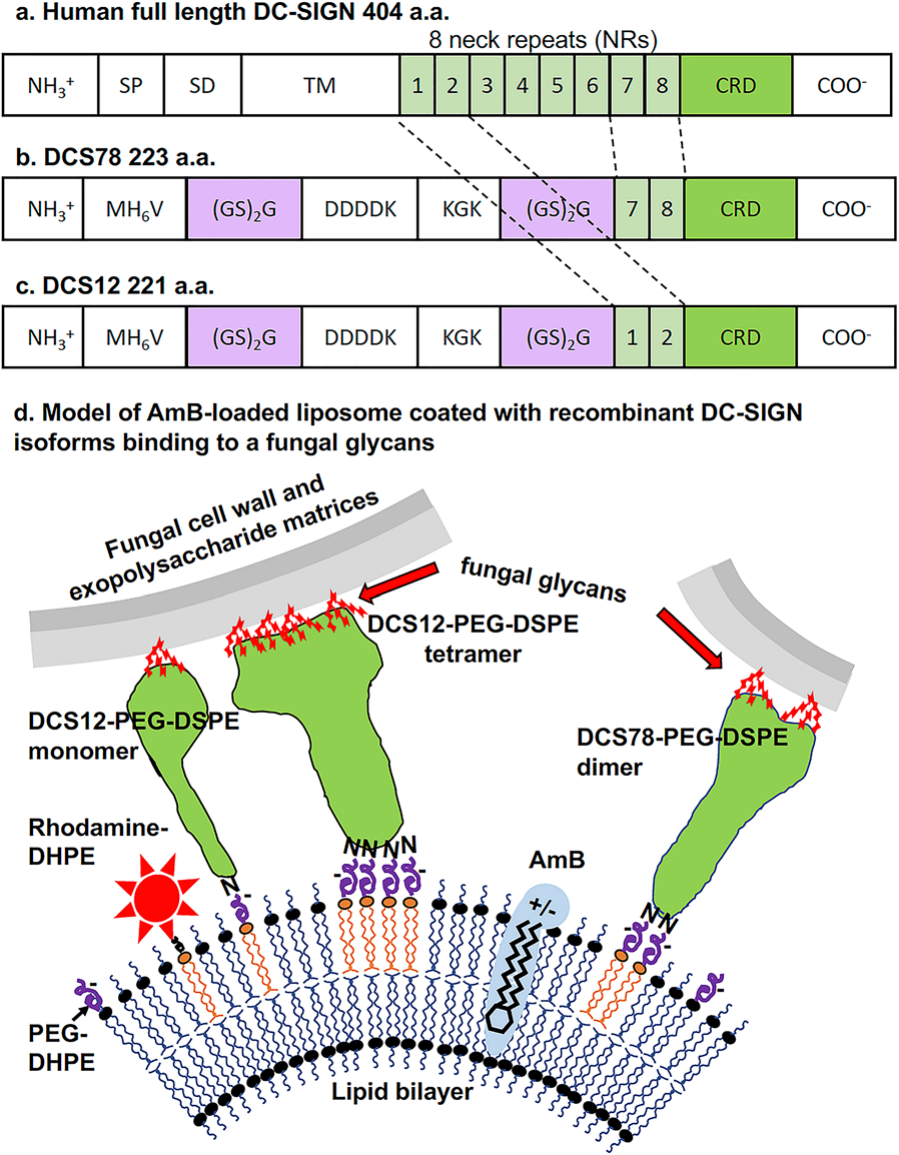
Recombinant human DC-SIGN isoforms were expressed, purified, and used to target drug loaded liposomes to fungal glycans. **a** Full length native human DC-SIGN is composed of a SP (Signal Peptide domain), SD (Signaling Domain), TM (Transmembrane domain), eight neck repeats (NRs), and a carbohydrate recognition domain (CRD). We expressed and purified two truncated recombinant isoforms of human DC-SIGN each with two NRs fused to the CRD (**b**) DCS78 contains NRs 7 and 8. **c** DCS12 contains NRs 1 and 2 and each contained a KGK sequence for coupling to a free K residue. Both DC-SIGN isoforms were used to target drug loaded liposomes. **d** Model of a targeted liposome. DCS12 and DCS78 monomers and predicted homodimers, and/or homotetramers (green irregular structures) have their shapes modeled from crystallographic structures of DC-SIGN monomers and multimers linked to two NRs [23]. They are shown binding to cognate glycans (e.g., red sugar moieties) in the cell walls and exopolysaccharide matrices of fungal cells. DCS12 and DCS78 were coupled to the lipid carrier DSPE-PEG via a lysine amino group (N) in each polypeptide. DHPE-Rhodamine (red star) along with DCS12-PEG-DSPE or DCS78-PEG-DSPE were inserted via their respective lipid moieties, DHPE and DSPE, into the liposomal membrane of amphotericin B loaded pegylated liposomes, AmB-LLs, to make red fluorescent DCS12-AmB-LLs and DCS78-AmB-LL. Each liposome contains approximately 3000 rhodamine molecules, 1500 monomers of a DC-SIGN isoform, and 16,500 AmB molecules [4]. DC-SIGN derived monomers are free to float in the membrane and are predicted to form functional multimers. The various objects in this diagram could not be drawn to the same scale due their extreme size differences. Single letter amino acid coding of particular domains are indicated: MH_6_V (hexa-histidine (H) tag for affinity purification), (GS)_2_G (flexible linkers to improve accessibility to flanking domains), DDDDK (protease processing site for potential removal of multimeric histidine tag), KGK (contains two lysine (K) residues for potential coupling to lipid carrier). Definitions: DSPE (1,2-Distearoyl-sn-glycero-3-phosphoethanolamine), DHPE (1,2-Dihexadecyl-sn-glycero-3-phosphoethanolamine)

Full-length human DC-SIGN (NCBI accession # Q9NNX6.1) is a 404 amino acid (aa) long protein (Fig. 1). The protein includes 8 neck repeats NR1 to NR8 (each 21 to 23 aa long) linked to its C-terminal CRD (151 aa) [23, 24] (Fig. 1a, Additional file 1: Fig. S1a). Alternate splicing produces a number of DC-SIGN isoforms that lack from one to seven of the eight NRs, but all isoforms retain the CRD [25]. The various isoforms of DC-SIGN float in the cell membrane as monomers, dimers, and tetramers and all three forms bind glycan ligands [24, 26–29]. Different combinations of NRs stabilize distinct homo-multimers which presumably have higher avidity than monomers and are proposed to provide alternate membrane positioning that may influence the specificity of binding to diverse glycans expressed by various pathogens [27, 30]. Herein we tested the ability of two recombinant isoforms of DC-SIGN to selectively target antifungal drug loaded liposomes to fungal pathogens. Liposomes targeted by one isoform in particular showed significantly enhanced binding and antifungal activity relative to untargeted antifungal liposomes.

## Results

### The design of DC-SIGN targeted liposomes

We engineered two recombinant isoforms of human DC-SIGN. Each isoform contains the CRD and two NRs (Fig. 1b, 1c, Additional file 2: Fig. S1b, c) and we produced them in mg quantities. They each lack the signal peptide domain (SP), signaling domain (SD) and transmembrane domain (TM) found in the native human DC-SIGN protein (Fig. 1a). The DCS78 (24.2 kDa) construct has the proximal NR7 and NR8 fused to the CRD. NR7 and NR8 are retained in most known mRNA splice variants [25] and all known human genetic polymorphisms [30], but neither splice variants nor polymorphic isoforms have been reported with just these two repeats alone. Hence, this is not a known native isoform. A recombinant isoform with only NR7 and NR8 fused to the CRD is known to form homodimers [30], but its glycan binding properties are unknown. The DCS12 (24.9 kDa) construct has distal NR1 and NR2 fused to the CRD (Fig. 1c). The design of DCS12 is based on a known human splice-variant encoded isoform of DC-SIGN that efficiently forms functional homotetramers and recognizes *C. albicans* cells, mannan-agarose, and *N*-acetyl galactosamine-agarose [25]. The DCS12 and DCS78 peptides were coupled to a lipid carrier and inserted into the membrane of amphotericin B loaded pegylated liposomes, AmB-LLs, to make DCS12-AmB-LLs and DCS78-AmB-LLs, respectively. The two modified DC-SIGN peptides are predicted to float in the liposome membrane and form active homomultimers that bind fungal glycans as modeled in Fig. 1d. We also coupled Bovine Serum Albumin (BSA, 65 kDa) to the same lipid carrier and constructed BSA coated BSA-AmB-LLs as a protein coated untargeted liposome control [4]. Rhodamine B was inserted quantitatively into the liposome membrane to fluorescently tag all four types of liposomes, AmB-LLs, DCS12-AmB-LLs, DCS78-AmB-LLs, and BSA-AmB-LLs. The chemical compositions of all four types of liposomes examined herein and for comparison that of AmBisome® are given in Additional file 1: Table S1.

### DCS12 and DCS78 coated liposomes bound to the fungal exopolysaccharide matrices of three diverse fungal species

**Fig. 2:**
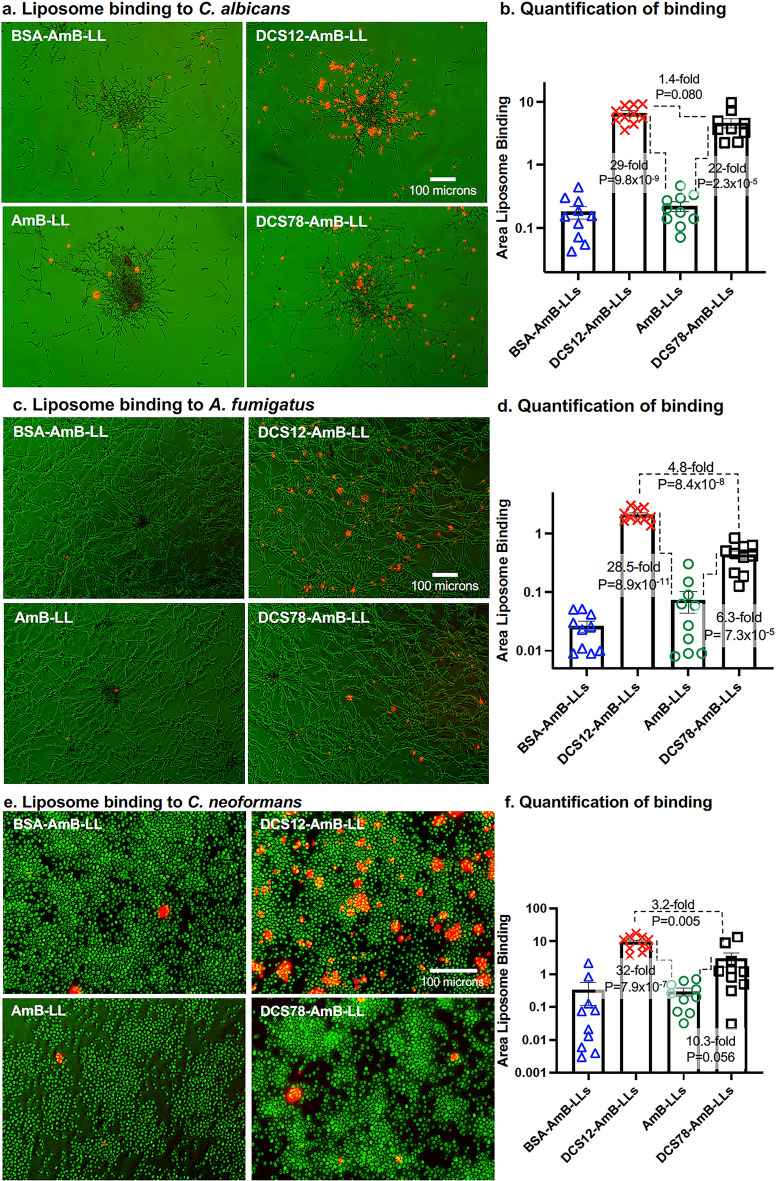
DC-SIGN targeted liposomes, DCS12-AmB-LLs and DCS78-AmB-LLs, bound significantly more efficiently to the exopolysaccharide matrices of three highly divergent fungal pathogens than untargeted AmB-LLs. **a**, **c**, **e** Representative photographic images of fluorescent liposomes binding to bright field images of fungal cells are presented. **a** *C. albicans* (×10 magnification)*. c* *A. fumigatus* (×10 magnification)*. e* *C. neoformans* (×20 magnification)*.* **b**, **d**, **f** The relative area of red fluorescent liposome binding (log_10_) was quantified as shown in scatter bar plots on the right. N = 10 for each bar. **f** The scale of the plot for *C. neoformans* had to be expanded from three to five logs to accommodate more widely distributed data. Standard errors and P values are indicted

We examined the ability of DCS12-AmB-LLs and DCS78-AmB-LLs to bind fungal cells in vitro and quantified these results by measuring the relative area of red fluorescent liposome binding to fungal cells as compared to that of AmB-LLs and BSA-AmB-LLs. Representative photographic images of red fluorescent liposomes binding to bright field images of fungal cells in green are presented in Fig. 2. Both DCS12-AmB-LLs and DCS78-AmB-LLs bound to what appears to be secreted patches of exopolysaccharide matrix surrounding colonies of *C. albicans* hyphae (Fig. 2a). Respectively, they bound at least 29-fold and 22-fold more strongly than AmB-LLs (P = 9.8 × 10^–9^, P = 2.3 × 10^–5^) (Fig. 2b). DCS12-AmB-LLs may have bound slightly more efficiently than DCS78-AmB-LLs (P = 0.08). Patches of exopolysaccharide binding appeared more frequent at the periphery of colonies rather than at the center. Respectively, DCS12-AmB-LLs and DCS78-AmB-LLs bound 28-fold (P = 8.9 × 10^–11^) and 6.3-fold (P = 7.3 × 10^–5^) more strongly to patches of exopolysaccharide associated with *A. fumigatus* hyphae than AmB-LLs (Fig. 2c, d). DCS12-AmB-LLs bound 4.8-fold more strongly than DCS78-AmB-LLs (P = 8.4 × 10^–8^). Respectively, DCS12-AmB-LLs and DCS78-AmB-LLs bound 32-fold (P = 7.9 × 10^–7^) and tenfold (P = 0.056) more strongly to the exopolysaccharide matrix allied with *C. neoformans* cells than AmB-LLs (Fig. 2e, f). DCS12-AmB-LLs bound threefold more strongly than DCS78-AmB-LLs (P = 0.005). BSA-AmB-LL control liposomes, did not bind noticeably stronger or weaker to any of the three fungal species than AmB-LLs, suggesting that simply coating liposomes with protein was not sufficient to enhance or diminish binding. Although we cannot exclude low levels of cell wall binding, neither of the DC-SIGN isoform-targeted liposomes appeared to be strongly associated with fungal cell walls. Because DCS12 coated liposomes bound to all three species more efficiently than DCS78 coated liposomes and represents a native isoform conformation, we focused on DCS12-AmB-LLs in the following experiments examining antifungal activity.

### DCS12-AmB-LLs inhibited or killed three fungal species in vitro more efficiently than control liposomes

**Fig. 3:**
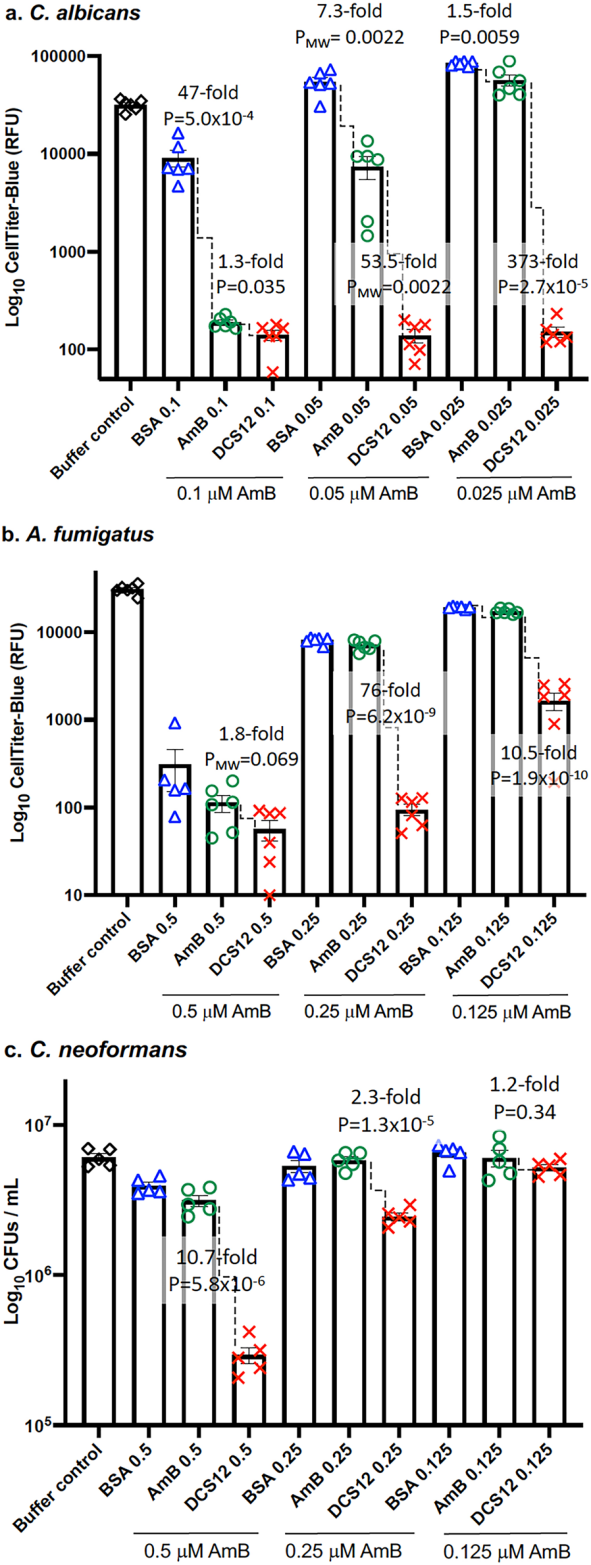
DCS12-AmB-LLs more efficiently inhibited or killed three diverse fungal species in vitro than un-targeted control liposomes. In vitro grown fungal cells were treated with DCS12-AmB-LLs, BSA-AmB-LLs or AmB-LLs delivering the indicated concentrations of AmB or with liposome dilution buffer. **a** *C. albicans* hyphal cells growing in RPMI in microtiter plates were treated with liposomes delivering 0.1, 0.05 and 0.025 µM AmB. Metabolic activity was measured as relative fluorescence units (RFU) generated from reduction of the CellTiter-Blue reagent. **b** *A. fumigatus* hyphal cells growing in RPMI in microtiter plates were treated with liposomes delivering 0.5, 0.25 and 0.125 µM AmB. Metabolic activity was measured with CellTiter-Blue reagent. **c** *C. neoformans* yeast cells growing in liquid YPD suspension were treated in with liposomes delivering 0.5, 0.25 and 0.125 µM AmB and surviving cells assayed as CFUs. N = 5 or N = 6 for each bar. Scatter bar plots indicate the inhibition and/or killing of fungal cells. The fold-difference in RFUs or CFUs and P or P_MW_ values are shown for informative comparisons

*Candida albicans* cells were grown in microtiter plates until they had reached the late germling and early hyphal stages. The cells were then treated for 1 h with AmB-LLs, BSA-AmB-LLs or DCS12-AmB-LLs delivering AmB at the indicated concentrations. Metabolic activity was measured with CellTiter-Blue (CTB) reagent. CTB assays measure mitochondrial reductase activity that is linked to active electron transport in living cells, and which generates a fluorescent product. Compared to uncoated AmB-LLs delivering the same concentrations of AmB, targeted DCS12-AmB-LLs delivering 0.05 and 0.025 µM AmB reduced the metabolic activity of *C. albicans* cells 53-fold (P = 0.0036) and 373-fold (P = 2.7 × 10^–5^) more efficiently, respectively (Fig. 3a). At a higher AmB concentration of 0.1 µM, AmB-LLs were much more effective at inhibiting metabolic activity. Hence at this concentration, DCS12-AmB-LLs were only slightly more effective than AmB-LLs (P = 0.035). Using even higher AmB concentrations of 0.2 or 0.4 µM and more cells per well, we could not observe any differences between the performance of AmB-LLs and DCS12-AmB-LLs (Additional file 4: Fig. S3b). BSA-AmB-LLs were less effective than AmB-LLs, suggesting that coating liposomes with a protein that did not target them to fungal cells may have interfered with antifungal liposome activity. Biological replicates of this experiment confirmed that DCS12-AmB-LLs delivering low concentrations of AmB (0.05 µM and 0.025 µM) performed significantly better than AmB-LLs (Additional file 4: Fig. S3a). The relatively low metabolic activity of cells in the Buffer treated control samples relative to some AmB-LL and BSA-AmB-LL samples (Fig. 3a and Additional file 4: Fig. S3a) may have resulted from the cells having reached stationary phase and becoming metabolically less active or because the fluorescent product resorufin was further reduced to non-fluorescent hydroresorufin [31].

*Aspergillus fumigatus* conidia were germinated and grown to the early germling stage in microtiter plates and then treated for 4 h with liposomes delivering the indicated concentrations of AmB. The CTB assays of metabolic activity after treatment are shown in Fig. 3b. Compared to AmB-LLs, DCS12-AmB-LLs delivering 0.25 and 0.125 µM AmB reduced the metabolic activity of *A. fumigatus* 76-fold (P = 6.2 × 10^–9^) and 10.5-fold (P = 1.9 × 10^–10^) more efficiently, respectively. Because AmB-LLs were more inhibitory when delivering a higher AmB concentration of 0.5 µM, we could not detect a significant benefit from targeting at this concentration. Biological replicate experiments confirmed the superior performance of DCS12 targeted liposomes (Additional file 4: Fig. S3c).

*Cryptococcus neoformans* cells were grown in rich liquid medium, treated for 4 h with AmB-loaded liposomes, and grown overnight. The surviving cells were diluted onto agar plates and assayed as colony forming units per mL of media (CFUs/mL). DCS12-AmB-LLs delivering 0.5 and 0.25 µM AmB reduced the number of *C. neoformans* CFUs/mL 10.7-fold (P = 5.8 × 10^–6^), 2.3-fold (P = 1.3 × 10^–5^), respectively, relative to AmB-LLs delivering the same concentrations of AmB (Fig. 3c). A biological replicate is shown in Additional file 4: Fig. S3.

### DCS12-AmB-LL dramatically reduced the fungal burden in mouse models of invasive candidiasis and pulmonary aspergillosis

**Fig. 4:**
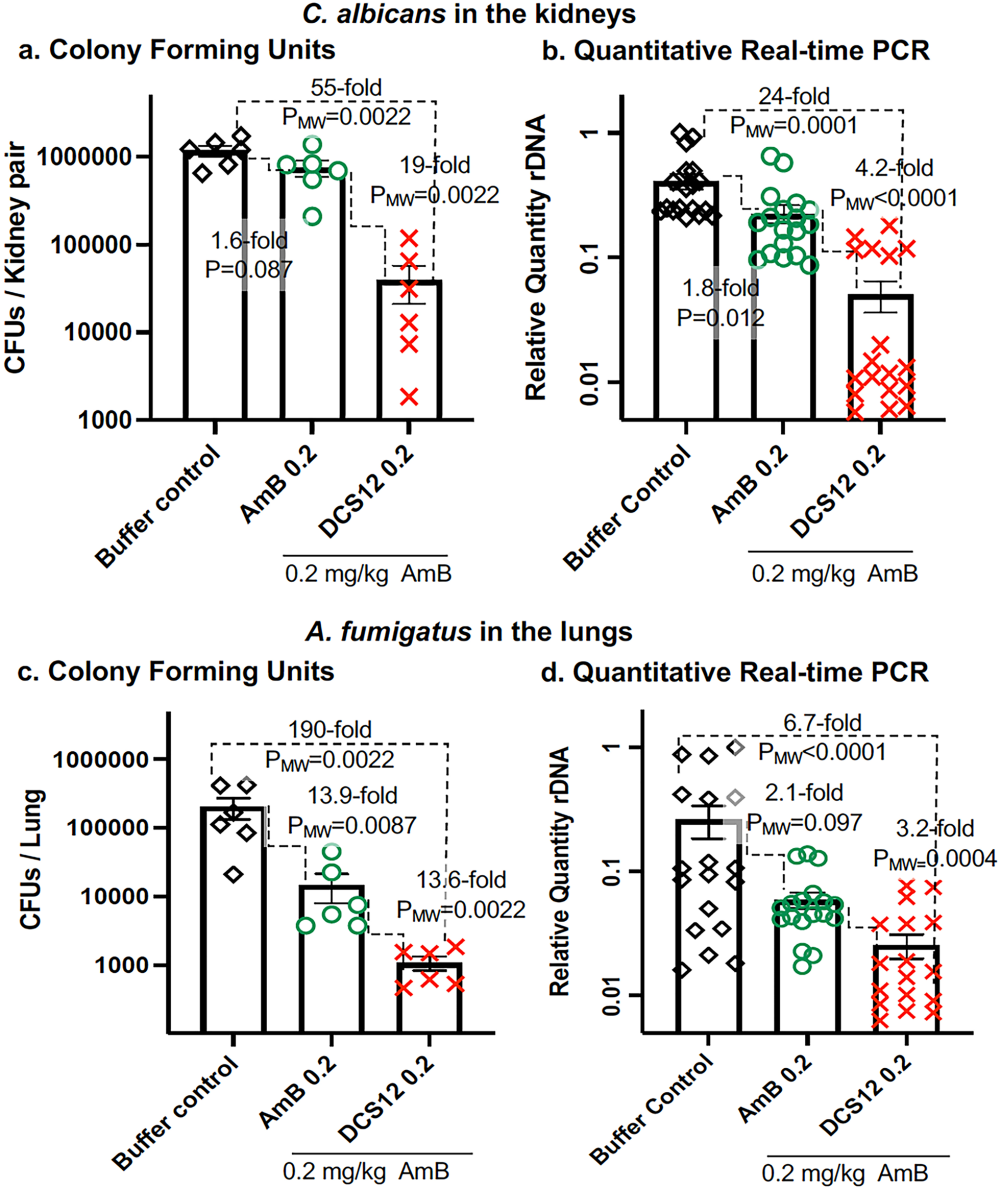
DCS12-AmB-LLs were significantly more effective at reducing the number of *C. albicans* cells in the kidneys and *A. fumigatus* in the lungs than AmB-LLs in mouse models of disease. Neutropenic mice with invasive candidiasis and pulmonary aspergillosis were treated with DCS12-AmB-LLs or AmB-LLs delivering 0.2 mg/kg AmB or with liposome dilution buffer. **a**, **b** Fungal burden of *C. albicans* in the kidneys. **c**, **d** Fungal burden of *A. fumigatus* in the lungs. **a**, **c** A scatter bar plot compares the average number of CFUs per mouse kidneys or lungs for the three treatment groups. Each mouse is represented by one data point. **b**, **d** The Relative Quantity (RQ) of fungal rDNA intergenic spacer (IGS) was determined by qPCR using species specific primers on parallel samples of kidney and lung homogenates from the same mice used to assay CFUs. Three replicates qPCR reactions were run on each sample. N = 6 mice for each treatment group. Standard errors are indicated. Fold differences and T.Test determined P values or Mann–Whitney determined P_MW_ values are shown for informative comparisons

In the candidiasis model, neutropenic mice were infected intravenously with *C. albicans* yeast cells and treated the same day with an intravenous injection of AmB-LLs or DCS12-AmB-LLs delivering 0.2 mg/kg AmB or control buffer. At 24 h post infection (PI), the mice were euthanized and their fungal burden in the kidneys was measured. Mice treated with DCS12-AmB-LLs showed a 19-fold lower average number of CFUs per kidney pair relative to AmB-LL treated mice (P_MW_ = 0.002, Fig. 4a) and 55-fold lower than buffer treated infected mice (P_MW_ = 0.002). Quantitative real time qPCR assays of the amount of fungal rDNA intergenic transcribe spacer (ITS) in duplicate samples revealed a 4.2-fold reduction in fungal burden in the DCS12-AmB-LLs group compared to AmB-LLs (P_MW_ < 0.0001, Fig. 4b) and a 24-fold reduction relative to buffer treated infected mice (P_MW_ = 0.0001). The differences between the results obtained from CFU and qPCR assays may be due to the contribution of DNA from dead cells in the DCS12-AmB-LL sample assayed by qPCR. AmB-LLs produced a marginally significant 1.6-fold and 1.8-fold reductions in fungal burden relative to the buffer treated infected controls (P = 0.087 to 0.012). Biological replicates of this experiment demonstrated the improved performance for DCS12-AmB-LLs over AmB-LLs (Additional file 5: Fig. S4a and b).

In the aspergillosis model, neutropenic mice were infected by the oropharyngeal delivery of *A. fumigatus* conidia and the next day given an oropharyngeal treatment with liposomes delivering 0.2 mg/kg AmB or control buffer. At 3 days PI, the mice were euthanized and their fungal burden in the lungs was measured. DCS12-AmB-LLs treated mice showed a 13.6-fold lower average number of CFUs per lung relative to AmB-LL treated mice (P_MW_ = 0.002, Fig. 4c) and 190-fold reduction relative to buffer treated infected mice (P_MW_ = 0.002). QPCR assays of rDNA ITS in duplicate samples revealed a 3.2-fold reduction in fungal burden in the DCS12-AmB-LLs relative to AmB-LLs treated mice (P_MW_ < 0.0004, Fig. 4d) and a 6.7-fold reduction relative to buffer treated mice (P_MW_ = 0.0001, Fig. 4d). At 0.2 mg/kg, AmB-LLs significantly reduced the number of CFUs relative to buffer treated control mice (P_MW_ = 0.0087), but the difference was not statistically significant when assayed by qPCR (P_MW_ = 0.097). A biological replicate is shown in Additional file 5: Fig. S4c and d.

## Discussion

We show that DC-SIGN is an effective liposome targeting protein. Alternate RNA splicing of DC-SIGN transcripts results in the expression of a variety of truncated membrane bound and soluble isoforms that combine the CRD with different numbers and combinations of neck repeats [25, 32]. Our results both complement and expand the ligand specificity determined by others using various isoforms by showing that truncated isoforms DCS12 and DCS78 bound to three diverse fungal species and that DCS12 showed superior fungal binding over DCS78. We speculate that NR1 and NR2 orient the CRD in DCS12-AmB-LLs in a more optimal conformation for binding fungal cognate ligands than provided by NR7 and NR8 in DCS78-AmB-LLs. The efficient binding of both isoforms to these three fungal pathogens gave us confidence that DC-SIGN had potential as an anti-infective liposome targeting protein. Because DCS12 is a natural human isoform, it should have low immunogenicity if used in the clinic.

The minimum inhibitory concentrations (MIC_50_) for commercial liposomal AmB (L-AmB, AmBisome®) against *Candida spp.*, *Aspergillus spp.,* and *Cryptococcus spp.,* which we will define as at least a 50% reduction in metabolic activity or in CFUs, varies modestly among previous publications and experimental designs. MIC_50s_ are typically 4 to 8 µM (3.7 to 7.4 mg/L) for in vitro grown cells of all three species [33–35] and 5 to 20 mg/kg to suppress fungal burden and significantly improve mouse survival in immune-suppressed mouse models of candidiasis and aspergillosis [34, 36, 37]. We showed that order of magnitude lower concentrations of AmB delivered as DCS12-AmB-LLs significantly reduced cell viability in vitro, concentrations at which AmB-LLs were relatively ineffective. The presence of metabolically inactive cells that resist high drug concentrations in biofilms (e.g., persister cells) may necessitate extended treatments in vivo [38]. Yet, we showed that single low doses of AmB delivered as DCS12-AmB-LL dramatically reduced fungal cell numbers in kidney and lungs and were significantly more effective than untargeted AmB-LLs at these concentrations. Lower effective doses of AmB should translate into greater efficacy and reduced antifungal drug toxicity in the clinic. In the future we will need to specifically examine the potential of DC-SIGN targeted antifungal liposomes against biofilms.

The present study of DC-SIGN-targeted antifungal liposomes is a continuation of our efforts to design DectiSomes for pan-antifungal therapies with increased efficacy [1–5]. We have previously shown that Dectin-1 and Dectin-2 are effective at targeting liposomes to fungal cells (1–5). Herein we showed that DC-SIGN targeting improved the performance of liposomal AmB against three highly divergent fungal species, whose common ancestry dates nearly to the divergence of the fungal kingdom from its animal and protist ancestries [21]. Goyal et al. [6] summarized what is known about 13 C-type lectin pathogen receptors that recognized one or more representative species from 20 distinct genera of fungal pathogens. In addition to the three genera of fungal pathogens represented herein, they reported that DC-SIGN also recognizes *Talaromyces spp.* (f.k.a., *Penicillium spp.*)*, Saccharomyces spp.,* and *Chrysosporium spp*., 6 of the 20 genera of pathogenic fungi [6]. Considering the evolutionary depth of fungal recognition by DC-SIGN, it is reasonable to propose that DC-SIGN coated liposomes might effectively deliver anti-infective drugs to life-threatening pathogens from other kingdoms, whose oligoglycans and lipoglycans are also recognized by DC-SIGN. For example, DC-SIGN binds cognate ligands expressed by the bacterium *Mycobacterium tuberculosis* [15], the animal helminth *Schistosoma mansoni* [39], and the protist *Leishmania infantum* [40] and hence may be effective at targeting lipid nanoparticle packaged anti-infectives to pathogens from these kingdoms. Respectively, these pathogens cause millions, hundreds of thousands, and tens of thousands of deaths annually and drug treatment failures are common for all three [41, 42]. It is worth noting that AmBisome® has been an FDA approved treatment for visceral leishmaniasis since 1997 [43].

In this study we had remotely intercalated the hydrophobic end of amphiphobic AmB into the bilipid membrane of preformed liposomes (Fig. 1). We have previously remotely incorporated the amphiphobic echinocandin antifungal, anidulafungin, into liposomes [2]. There are a variety of methods for remote loading of both hydrophilic and hydrophobic drugs into liposomes [44]. Remote loading methods have allowed us to work on smaller scale than older organo-chemical methods, which for example, may involve flash evaporators. However, newer microfluidic methods are scalable and microfluidics simplifies and speeds up the assembly of various types of lipid nanoparticles from constituent lipids and almost any class of drug beyond remote loading methods [45, 46]. Indeed Pfizer’s and Moderna’s *Spike mRNA* lipid nanoparticle vaccines are both produced using microfluidic methods [47]. In the future we plan to construct DectiSomes using microfluidics.

The AmB-LLs employed herein are pegylated liposomes in which 5% of their constituent lipids are the pegylated lipid mPEG2000-DSPE (Additional file 1: Table S1), wherein the partially negatively charged hydroxyl rich PEG group is proposed to be on the outside of the liposomes in the aqueous environment (Fig. 1). The first amphotericin B loaded lipid nanoparticle drugs, including AmBisome®, were approved for clinical use in the mid- to late 1990s and were not pegylated [48], likely because the benefits of pegylated nanoparticles [49, 50] were not yet fully appreciated. Pegylated liposomes are protected from opsonization and phagocytosis [51] and for this reason are called stealth liposomes [52]. The half-lives of packaged drugs are extended in pegylated liposomes. We recently demonstrated that our pegylated AmB-LLs significantly outperform AmBisome® in a mouse model of disseminated candidiasis [2], presumably because pegylation extends the effective half-life of the liposomal AmB. In addition, we also have shown that the Dectin targeting of AmBisome® improves its performance in the mouse candidiasis model [2], generalizing the benefits of targeting to other lipid nanoparticle preparations.

## Conclusions

DCS12 is a truncated recombinant isoform of DC-SIGN constructed from part of a natural full-length DC-SIGN isoform, while DCS78 is part of a truncated recombinant isoform with no known homolog in nature. Compared to untargeted liposomes, DCS12-AmB-LLs and DCS78-AmB-LLs bound at significantly higher levels to the exopolysaccharide matrices associated with *C. albicans, A. fumigatus,* and *C. neoformans* grown in vitro, with DCS12-AmB-LLs showing superior binding activity. DCS12-AmB-LLs were far more effective at inhibiting and/or killing all three fungal species in vitro than AmB-LLs or BSA-AmB-LLs. In neutropenic mouse models of invasive candidiasis and pulmonary aspergillosis, DCS12-AmB-LLs were significantly more effective in reducing fungal burden in the kidneys and lungs than untargeted AmB-LLs. By dramatically lowering the effective dose of antifungal drug, DCS12-AmB-LLs have the potential to overcome dose dependent drug resistance and perhaps persister-like cells in biofilms. DC-SIGN is the third C-type lectin we’ve used to construct DectiSomes that had improved liposomal drug performance. Collectively, these results begin to generalize the benefits of targeting anti-infective liposomes to diverse pathogenic cells using pathogen receptor CRDs. DC-SIGN-targeted anti-infective drug-loaded liposomes may also be effective at treating pathogens in the bacterial, animal, and protist kingdoms that express DC-SIGN’s cognate ligands.

## Methods

### Aim, design, and study setting

The aim of this study was to show that DectiSomes coated with the CRD and two NRs of DC-SIGN targeted anti-infective loaded liposomes to diverse fungal species. Based on the structures modeled for DC-SIGN monomers and multimers, we predicted that two NRs might optimally position the CRD for glycan binding by liposomes as illustrated in Fig. 1d [23]. Drug loaded liposomes were coated with recombinant isoforms of DC-SIGN and used to treat fungal cells. Quantitative binding assays, metabolic and growth assays in vitro, and fungal burden assays in vivo in mouse models of fungal diseases were employed to examine efficacy.

### Strains of fungi and medium

Three fungal species were examined herein and grown on polystyrene 24- (Costar) or 96-well (BD Falcon) microtiter plates without agitation or grown in liquid suspension with agitation. *C. albicans* strain CA14 [53] derived from a human isolate (SC5314, ATCC MYA-2876) that was subsequently deleted for *URA3* (strain CA14, Δ*ura3*::imm434/Δ*ura3*::434) [54] and *A. fumigatus* A1163 were grown in RPMI 1640 medium with no phenol red pH indicator (Sigma #R8755) + 0.5% BSA (Sigma #A7906) or + 10% fetal bovine serum (Life Technologies GIBCO #16170-078) at 37 °C. The preparation of *A. fumigatus* conidia was described previously [4]. *C. neoformans* clinical isolate H99 [55] was grown in YPD (1% yeast extract, 2% peptone, 2% dextrose) with 0.5% BSA at 32 °C. H99 grows better in vitro at 32 °C than at 37 °C.

### Mice

Seven- to eight-week-old outbred female CD1 (CD-1 IGS) Swiss mice (27 g to 30 g ea.) were obtained from Charles River Labs. Six mice were housed per cage, given 12 h light and 12 h dark per day, maintained on a standard 20% protein diet (PicoLab® Rodent diet 20, LabDiet #5053), and given water liberally. Mice were rendered neutropenic by treating them 3 days prior to infection with 150 mg/kg of the antimetabolite cyclophosphamide and 75 mg/kg of the corticosteroid triamcinolone [5]. Mice were maintained in UGA’s Animal Care Facility.

### Preparation of DC-SIGN isoforms and integrating them into AmB loaded liposomes

The carboxyterminal extracellular CRD of DC-SIGN is hydrophobic and thus relatively insoluble in normal biological buffers. Soluble functional isoforms are inefficiently recovered from *E. coli.* For example, it was reported that only 0.5 to 0.7 mg of soluble DC-SIGN was recovered from a liter of isopropyl β-d-1-thiogalactopyranoside (IPTG) induced *E. coli* cultures in one of the best previous studies [24]. DCS12 and DCS78 polypeptides are predicted to be unstable and insoluble hydrophobic proteins (https://web.expasy.org/protparam/). Here we applied the same technologies that we used to overcome such difficulties in manipulating Dectin-1 and Dectin-2 [3, 4] to obtain modest quantities of DCS12 and DCS78 as summarized briefly here. *E. coli* optimized DNA encoding sequences were cloned into expression plasmid pET-45b + and transformed into *E. coli* strain BL21. Following 4 h induction with IPTG, the protein was solubilized and extracted from cell pellets in a 6 M Guanidine hydrochloride (GuHCl) buffer and affinity purified on nickel affinity resin. Approximately 40 mg of 75 to 80% pure DCS12 and DCS78 peptides were recovered (Additional file 3: Fig. S2). While still in a 6 M GuHCl buffer, DCS12 and DCS78 were coupled with the lipid carrier DSPE-PEG-3400-NHS. Gel exclusion chromatography over Bio-Gel P6 acrylamide resin was used to remove GuHCl and excess hydrolyzed coupling agent and to buffer exchange the protein into a 1 M arginine crowding buffer with beta-mercaptoethanol (BME) [4]. BSA was coupled via a native lysine amino group to DSPE-PEG-3400-NHS.

AmB-LLs were prepared as described previously by the remote loading of AmB into 100 nm diameter FormuMax liposomes (F10203, Plain) [4]. AmB-LLs are pegylated analogs of commercial unpegylated AmBisome® (a.k.a. L-AmB), with an equivalent amount of AmB, 11 moles percent relative to moles of liposomal membrane lipids, but a different lipid profile (Additional file 1: Table S1). The modified DCS12-PEG-DSPE and DCS78-PEG-DSPE proteins remained soluble in the 1 M arginine buffer and were integrated via their DSPE lipid moieties into the phospholipid bilayer membrane of AmB-LL at 1.0 mol percent relative to moles of liposomal lipid to make DCS12-AmB-LLs and DCS78-AmB-LLs (Additional file 1: Table S1). As a protein coated control, we made liposomes coated with 0.33 moles percent bovine serum albumin, BSA-AmB-LLs. We reduced the molar amount of BSA (65 kDa), because it is about three times larger than DCS12 (24.9 kDa) or DCS78 (24.2 kDa), and thus BSA-AmB-LLs are coated with an equivalent µg amount of protein (Additional file 1: Table S1). Lissamine rhodamine B-DHPE was simultaneously incorporated at two moles percent in all liposome preparations (Fig. 1d), allowing sensitive and quantitative comparisons of fluorescence to estimate the binding efficiency of all four liposomal preparations to fungal cells. DCS12-AmB-LL and DCS78-AmB-LL stocks were stored in the 1 M arginine buffer [4] such that the AmB concentration ranged from 600 to 800 µM. Fresh BME was added to 0.1 mM every month during storage. Liposomes remained active in their binding activity for several months when stored at 4 °C. Just prior to their use, DCS12 and DCS78 liposomal stocks were diluted with LDB2 buffer (20 mM HEPES, 10 mM triethanolamine, 150 mM NaCl, 10 mM CaCl_2_, fresh 1 mM beta-mercaptoethanol (BME), pH 8.0) or with growth medium. None of the liposome preparations examined herein aggregate significantly upon dilution into liposome dilution buffer or into growth medium.

### Microscopy and quantification of liposome binding to fungal cells

Cells to be photographed were grown in 24-well microtiter plates, washed thrice with phosphate buffered saline (PBS), fixed in 4% formaldehyde in PBS for 60 min, washed thrice, and stored at 4 °C in PBS. Fixed fungal cells were pre-incubated for 30 to 60 min with in LDB2 + 5% BSA at 23 °C. Liposomal stocks were freshly diluted approximately 100-fold into LDB2 + 5% BSA on the same day they were to be used and then incubating with cells such that the DCS12 or DCS78 or BSA protein concentration was approximately 1.0 µg/100 µL. After 1 h incubation at 23 °C, unbound liposomes were washed out with 4 changes of LDB2 + 5% BSA. Ten separate fluorescent images of rhodamine red fluorescent liposomes bound to cells grown on 24 well microtiter plates were taken at 10× or 20× magnification on an Olympus inverted microscope (Model IX70) with a digital camera. The relative area of red fluorescent liposome binding from 10 random images was quantified by taking an 8-bit grey-scale copies of the unmodified red fluorescent JPEG images into Image J (imagej.nih.gov/ij) as described previously for Dectin-2 coated liposomes [3]. The green channel of bright field images showing fungal cells and the red fluorescent images of liposomes were merged in Photoshop and enhanced for image presentation.

### Growth inhibition and viability assays following liposome treatment

Cell viability and metabolic activity after AmB loaded liposome treatment was assayed by a protocol modified from that described previously [3]. *C. albicans* yeast cells grown in YPD were inoculated at 4000 cells per well in 96 well microtiter plates in RPMI and grown for 7 h at 37 °C. Liposomal stocks were freshly diluted 1000-fold or more into fresh LDB2 buffer + 0.5% BSA and then 1:11 into growth medium to achieve the indicated final AmB concentrations. Control cells received an equivalent amount of buffer. After 1 h incubation at 37 °C, the plates were gently agitated for a few seconds and unbound liposomes were removed without further washing. 100 µL of fresh RPMI growth medium was added. After 4.5 h incubation at 37 °C, 20 µL of blue non-fluorescent CTB reagent resazurin (Promega Corp.) was added per well as per the manufacturer’s instructions. After 1.5 h incubation at 37 °C the fluorescent product resorufin was analyzed in a Bio-Tek Synergy HT fluorescent microtiter plate reader (ex485/em590). The fluorescent background from control wells with reagent, but no cells, was subtracted from readings of experimental wells. Data from six wells were averaged for each data point. CTB assays on *A. fumigatus* cells were performed using the same protocol in RPMI medium, except that 5000 conidia per well were grown for 8 h until they reached early germling stage, treated with liposomes for 4 h, washed once, grown overnight in fresh medium, and then examined for reductase activity.

In our hands CTB assays do not work for *C. neoformans* [3]. Therefore, we assayed growth inhibition and killing as percentage of surviving colony forming units (CFUs) after treatment in liquid medium. A fresh overnight culture of *C. neoformans* cells grown in YPD was diluted to 5000 cells /mL, grown for 3 h with shaking at 200 rpm, treated with liposomes delivering the indicated concentrations of AmB for 4 h with shaking, washed once, and grown in fresh medium overnight. The cells were diluted, plated on YPD agar, and the number of CFUs/mL of culture medium recorded.

### Fungal burden measurement

Neutropenic mice were infected intravenously with 7.5 × 10^6^ *C. albicans* yeast cells in 100 µL of PBS or by the oropharyngeal delivery of 1.0 × 10^6^ *A. fumigatus* conidia in 50 µL on Day zero. In the candidiasis model, mice were given an intravenous treatment 3 h PI with AmB-loaded liposomes. Fungal burden at 24 h PI in excised homogenized kidney pairs was estimated. In the pulmonary aspergillosis model, mice were given an oropharyngeal liposomal drug treatment at 20 h PI. Fungal burden in excised and homogenized lungs was estimated at day 3 PI. Organs were weighed and minced into hundreds of approximately 1 mm^3^ pieces, the pieces mixed to account for the uneven distribution of infection sites, and aliquoted into 25 mg samples. For CFUs assay, 25 mg of the minced kidney or lung tissue was homogenized for 60 s in 200 µL of PBS using a hand-held battery powered homogenizer (Kimble, cat#749540-0000) and blue plastic pestle (Kimble Cat#749521-1500). The homogenates were spread on YPD agar plates containing 100 µg/mL each of Kanamycin and Ampicillin, and CFUs were counted [2, 5]. For qPCR assays, DNA was extracted from parallel 25 mg samples of kidney and lung homogenates using Qiagen’s DNeasy® Blood & Tissue Kit (#69504) protocol modified with a bead beater step as we described previously [5]. qPCR was used to estimate the relative quantity of *rDNA* ITS in 100 ng samples of infected organ DNA using the conditions and species-specific primers described previously [2, 5].

### Data management

Data were recorded and managed in Excel (v. 16.16.27). Scatter bar plots were prepared and standard errors estimated in Graph Pad Prism 9 (v. 9.0.0). Most of the data were normally distributed, so the student’s two-tailed t test, T.TEST in Excel, was used to estimate P values. In those cases where the data for at least one sample in a comparison appeared to be non-parametric in their distribution, P values were estimated using the Mann–Whitney test in Prism 9 and were indicated as P_MW_ values.

## Supplementary Information

**Additional file 1: Table S1.** Liposome composition compared to that of Gilead’s AmBisome. https://www.astellas.us/docs/ambisome

**Additional file 2: Fig. S1**. Amino acid sequences of human DC-SIGN, DCS12 and DCS78. **a** Annotated amino acid (a.a.) sequence of full-length human DC-SIGN (CD209 Q9NNX6.1, 404 a.a.). **b** Annotated a.a. sequence of recombinant isoform DCS78*.* **c.** Annotated a.a. sequence of recombinant isoform DCS12*.*

**Additional file 3: Fig S2.** SDS PAGE analysis of affinity purified DCS12 and DCS78 polypeptides, before and after coupling to DSPE-PEG-NHS. Samples were resolved on a 12% polyacrylamide gel and stained with Coomassie Blue. Color coded molecular weight markers visible before Coomassie staining were tagged by poking carbon particles into the gel with a needle. Their sizes are indicated in kilo-Daltons (kDa). PEG is extremely hydrophilic, alters protein migration, and reduces the efficiency of Coomassie protein staining of hydrophobic protein domains.

**Additional file 4: Fig. S3.** Replicates of experiments in Fig. 3 showing the inhibition or killing of three fungal species by DCS12-AmB-LLs and AmB-LLs delivering various AmB concentrations in vitro. **a** A biological replicate of Fig. 3a. Wells of a microtiter plate were inoculated with 4000 *C. albicans* yeast cells per well. Cells were grown to late germling and early hyphal stages and treated for 60 min with liposomes delivering 0.1, 0.05 and 0.025 µM AmB. For experimental details see legend to Fig. 3 and Material and Methods. **b** This experiment was conducted and analyzed by methods similar to those described in Fig. 3a and Additional file 4: Fig. S3a except that plates were inoculated with 40,000 cells per microtiter well instead of 4000 cells/well, higher concentrations of AmB were explored, and cells were only grown for 3 h after liposomes were removed and just before adding CTB reagent. **c** Inhibition and killing of *A. fumigatus* grown in microtiter plates seeded with 4,500 conidia per well. **d.** Inhibition and killing of *C. neoformans* grow in liquid. Standard errors are indicated with a bar and whisker. N = 5 or N = 6 for each bar. Fold differences and P or P_MW_ values for comparisons between DCS12-AmB-LL and AmB-LL treated fungi are indicated.

**Additional file 5: Fig. S4.** Replicate experiments showing DCS12-AmB-LLs were significantly more effective at reducing the number or viability of *C. albicans* in the kidneys and *A. fumigatus* in the lungs than AmB-LLs in two distinct mouse models of disease (see Fig. 4). Neutropenic mice with invasive candidiasis and pulmonary aspergillosis were treated with DCS12-AmB-LLs or AmB-LLs delivering 0.2 mg/kg AmB or with liposome dilution buffer. **a**, **b** Fungal burden of *C. albicans* in the kidneys. **c**, **d** Fungal burden of *A. fumigatus* in the lungs. **a**, **c** Scatter bar plots compares the average number of CFUs per kidney pair or lung pair for the three treatment groups. Each mouse is represented by one data point. **b**, **d** The Relative Quantity (RQ) of fungal rDNA intergenic spacer (IGS) was determined by qPCR using species specific primers on parallel samples of kidney and lung homogenates from the same mice used to assay CFUs. Three replicates qPCR reactions were run on each sample. N = 6 mice for each treatment group. Standard errors are indicated. Fold differences and T.Test determined P values or Mann–Whitney determined P_MW_ values are shown.

## Data Availability

All new data discussed are presented within this publication and any data obtained from other publications were appropriately cited.

## Abbreviations

DC-SIGN: Dendritic Cell-Specific ICAM-3-Grabbing Non-Integrin 1, DC-SIGN1, CD-SIGN, CD209)
AmB: Amphotericin B
CRD: Carbohydrate recognition domain
qPCR: Quantitative real time PCR
CFU: Colony forming units
GuHCl: Guanidine hydrochloride
