# Supplementary material for: DC-SIGN targets amphotericin B-loaded liposomes to diverse pathogenic fungi
Source: Fungal Biol Biotechnol. 2021 Dec 24;8:22. doi: 10.1186/s40694-021-00126-3 (PMC8709943; doi:10.1186/s40694-021-00126-3)
Supplement: Supplementary file 1 — Additional file 1: Table S1. Liposome composition compared to that of Gilead’s AmBisome. https://www.astellas.us/docs/ambisome [file 40694_2021_126_MOESM1_ESM.docx]

**Additional Table S1. Liposome composition compared to that of Gilead’s AmBisome®**

| Compounds | Gilead’s^1^ AmBisome® also L-AmB | AmB-LLs | BSA-AmB-LLs | DCS12-AmB-LLs or DCS78-AmB-LLs |
| --- | --- | --- | --- | --- |
| Moles percent additions relative to moles of lipids in base liposomes |  |  |  |  |
| DCS12 or DCS78 or BSA | 0.0 | 0.0 | 0.33 | 1.0 |
| Amphotericin B | 10.6 | 11.0 | 11.0 | 11.0 |
| Lissamine Rhodamine-PE | 0.00 | 2.0 | 2.0 | 2.0 |
|  |  |  |  |  |
| Lipids of base liposomes defined as 100% |  |  |  |  |
| alpha-Tocopherol (form of Vitamine E) | 0.0003 | 0.0 | 0.0 | 0.0 |
| mPEG2000-DSPE (N-(Carbonyl-methoxypolyethylene glycol 2000)-distearoyl-glycerophosphoethanolamine ) | 0.0 | 5.0 | 5.0 | 5.0 |
| HSPC (soy phosphotidylcholaine) | 52.7 | 0.0 | 0.0 | 0.0 |
| DSPC (1,2-Distearol-sn-glycerol-3-phosphocholine) |  | 50.0 | 50.0 | 50.0 |
| DSPG (1,2-Distearoyl-sn-glycero-3-phosphoglycerol) | 21.1 | 0.0 | 0.0 | 0.0 |
| CHOL (Cholesterol) | 26.2 | 45.0 | 45.0 | 45.0 |
| Total unconjugated lipid in base liposomes = 100% | 100 | 100.0 | 100.0 | 100.0 |

1. Gilead’s AmBisome® (amphotericin B) liposome for injection. https://www.astellas.us/docs/ambisome.pdf
