# Supplementary figures and images for: DC-SIGN targets amphotericin B-loaded liposomes to diverse pathogenic fungi
Source: Fungal Biol Biotechnol. 2021 Dec 24;8:22. doi: 10.1186/s40694-021-00126-3 (PMC8709943; doi:10.1186/s40694-021-00126-3)

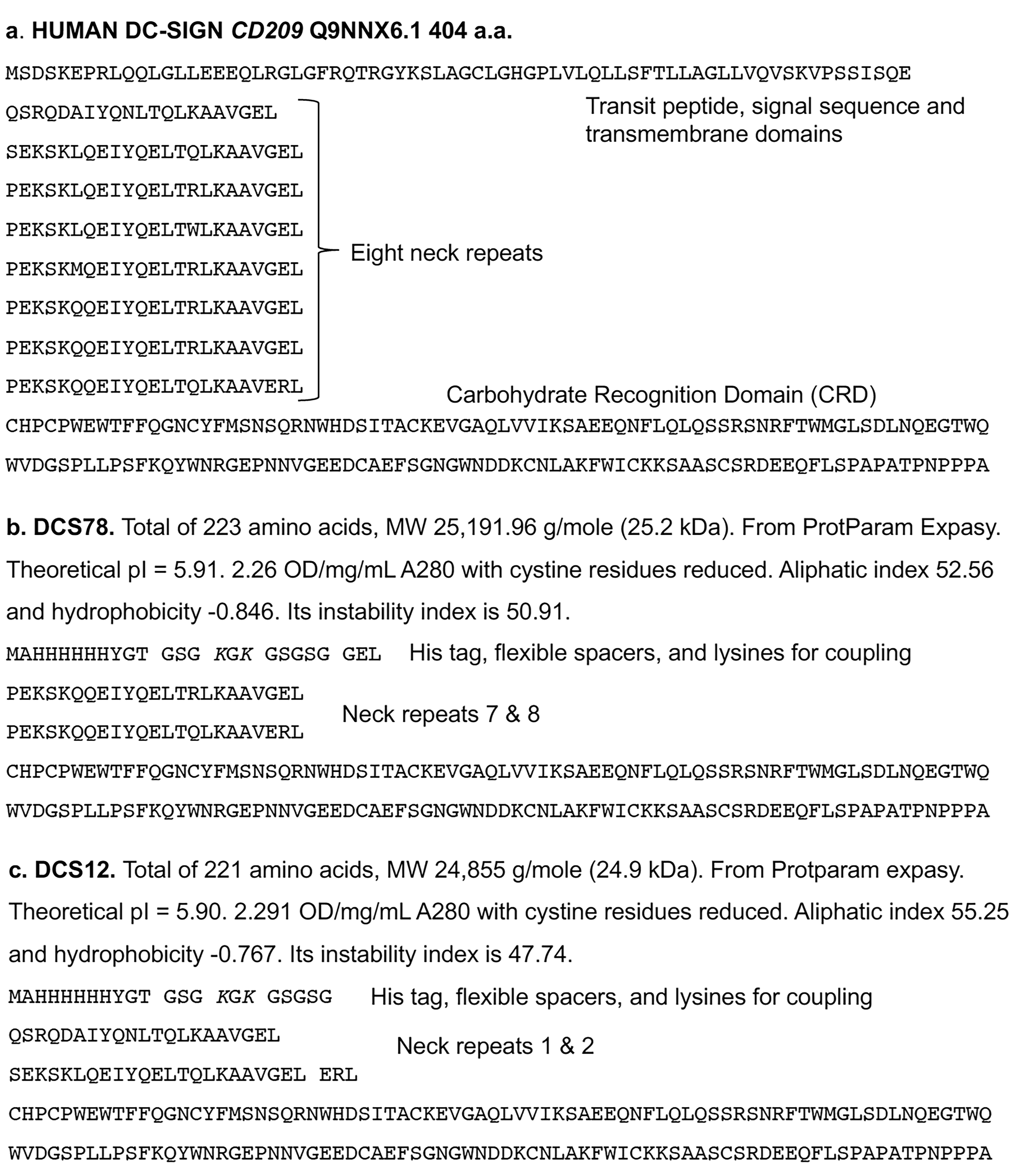

Supplement: Supplementary file 2 — Additional file 2: Fig. S1. Amino acid sequences of human DC-SIGN, DCS12 and DCS78. a Annotated amino acid (a.a.) sequence of full-length human DC-SIGN (CD209 Q9NNX6.1, 404 a.a.). b Annotated a.a. sequence of recombinant isoform DCS78. c. Annotated a.a. sequence of recombinant isoform DCS12. [file 40694_2021_126_MOESM2_ESM.jpg]

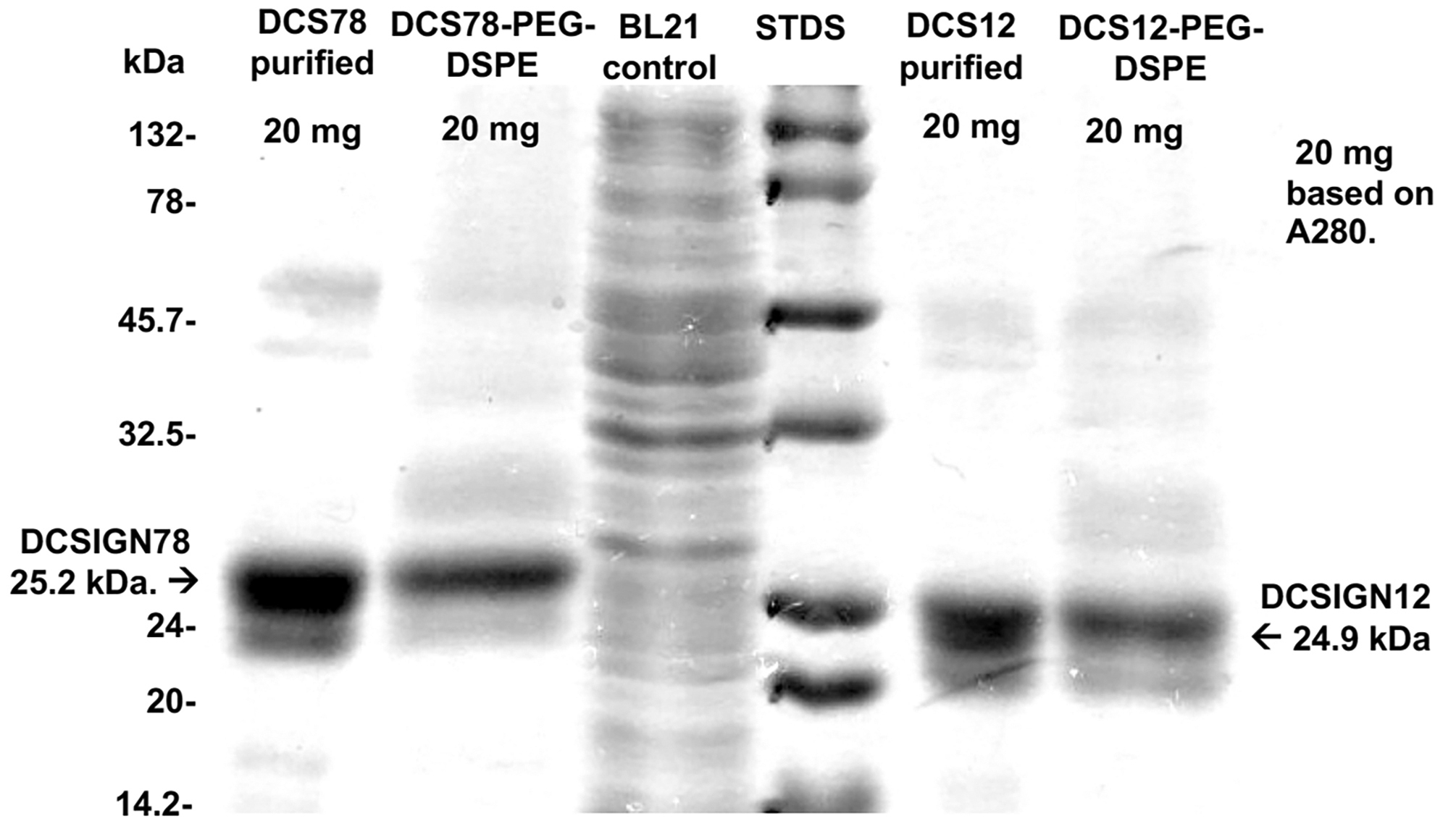

Supplement: Supplementary file 3 — Additional file 3: Fig S2. SDS PAGE analysis of affinity purified DCS12 and DCS78 polypeptides, before and after coupling to DSPE-PEG-NHS. Samples were resolved on a 12% polyacrylamide gel and stained with Coomassie Blue. Color coded molecular weight markers visible before Coomassie staining were tagged by poking carbon particles into the gel with a needle. Their sizes are indicated in kilo-Daltons (kDa). PEG is extremely hydrophilic, alters protein migration, and reduces the efficiency of Coomassie protein staining of hydrophobic protein domains. [file 40694_2021_126_MOESM3_ESM.jpg]

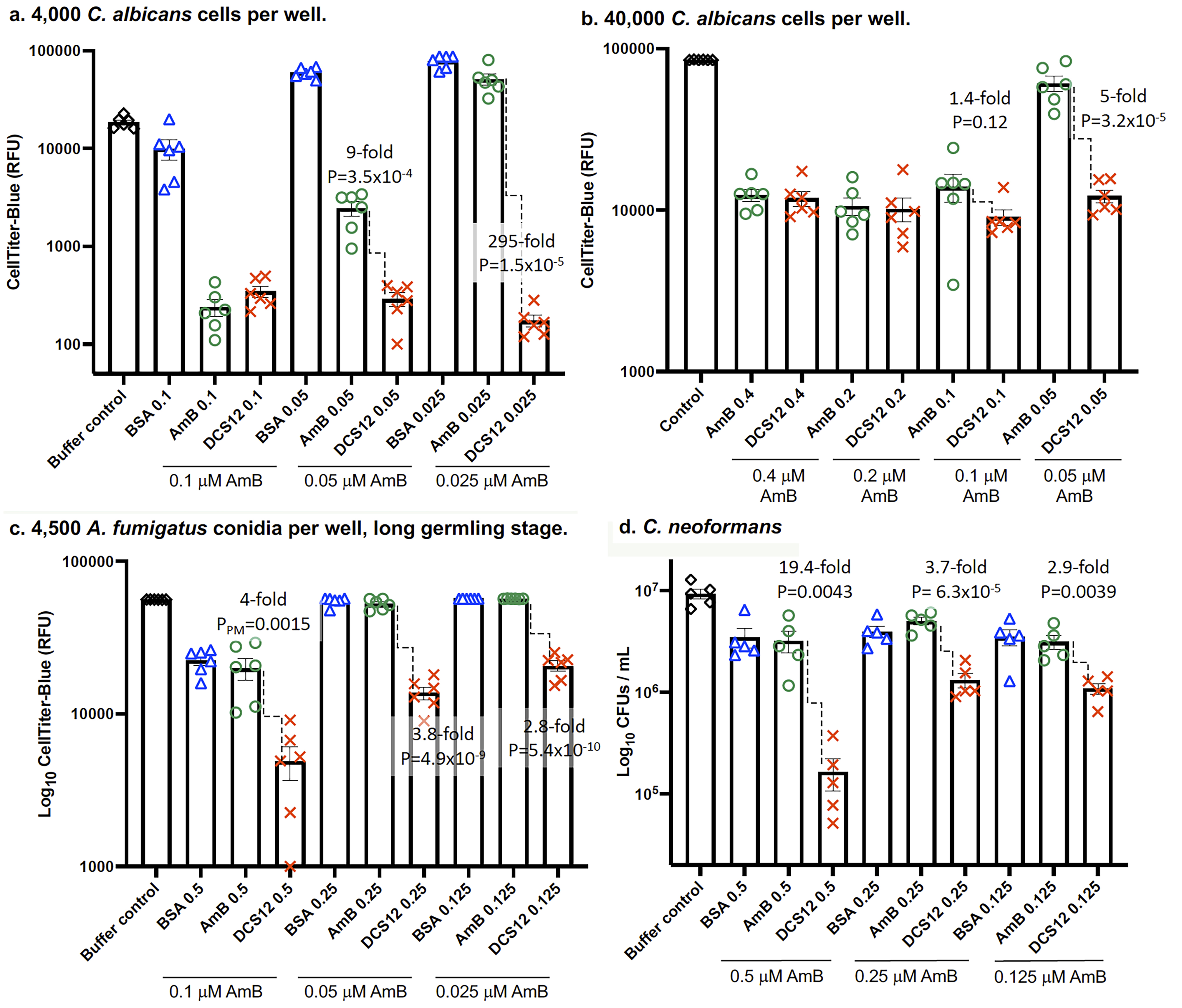

Supplement: Supplementary file 4 — Additional file 4: Fig. S3. Replicates of experiments in Fig. 3 showing the inhibition or killing of three fungal species by DCS12-AmB-LLs and AmB-LLs delivering various AmB concentrations in vitro. a A biological replicate of Fig. 3a. Wells of a microtiter plate were inoculated with 4000 C. albicans yeast cells per well. Cells were grown to late germling and early hyphal stages and treated for 60 min with liposomes delivering 0.1, 0.05 and 0.025 µM AmB. For experimental details see legend to Fig. 3 and Material and Methods. b This experiment was conducted and analyzed by methods similar to those described in Fig. 3a and Additional file 4: Fig. S3a except that plates were inoculated with 40,000 cells per microtiter well instead of 4000 cells/well, higher concentrations of AmB were explored, and cells were only grown for 3 h after liposomes were removed and just before adding CTB reagent. c Inhibition and killing of A. fumigatus grown in microtiter plates seeded with 4,500 conidia per well. d. Inhibition and killing of C. neoformans grow in liquid. Standard errors are indicated with a bar and whisker. N = 5 or N = 6 for each bar. Fold differences and P or PMW values for comparisons between DCS12-AmB-LL and AmB-LL treated fungi are indicated. [file 40694_2021_126_MOESM4_ESM.jpg]

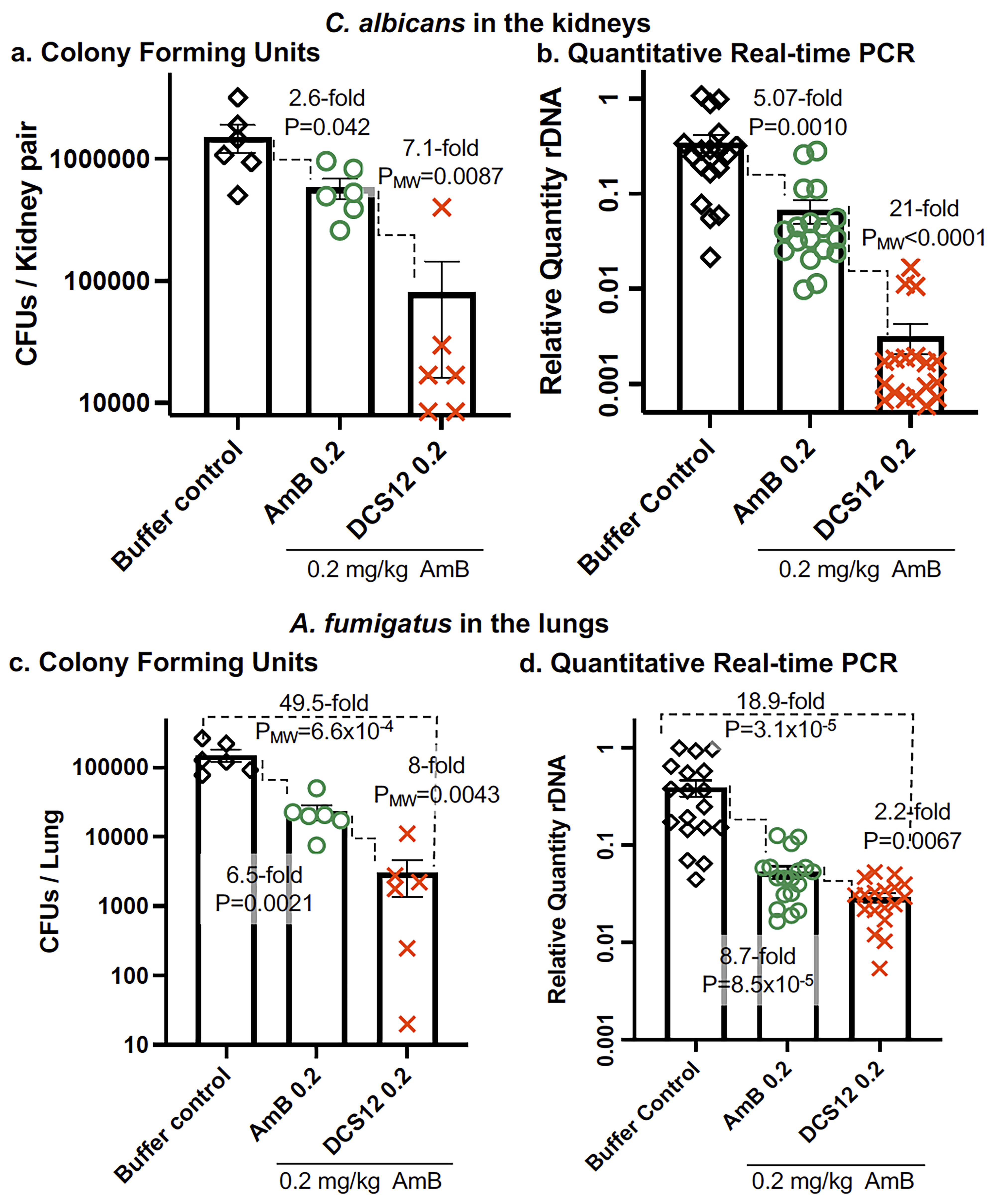

Supplement: Supplementary file 5 — Additional file 5: Fig. S4. Replicate experiments showing DCS12-AmB-LLs were significantly more effective at reducing the number or viability of C. albicans in the kidneys and A. fumigatus in the lungs than AmB-LLs in two distinct mouse models of disease (see Fig. 4). Neutropenic mice with invasive candidiasis and pulmonary aspergillosis were treated with DCS12-AmB-LLs or AmB-LLs delivering 0.2 mg/kg AmB or with liposome dilution buffer. a, b Fungal burden of C. albicans in the kidneys. c, d Fungal burden of A. fumigatus in the lungs. a, c Scatter bar plots compares the average number of CFUs per kidney pair or lung pair for the three treatment groups. Each mouse is represented by one data point. b, d The Relative Quantity (RQ) of fungal rDNA intergenic spacer (IGS) was determined by qPCR using species specific primers on parallel samples of kidney and lung homogenates from the same mice used to assay CFUs. Three replicates qPCR reactions were run on each sample. N = 6 mice for each treatment group. Standard errors are indicated. Fold differences and T.Test determined P values or Mann–Whitney determined PMW values are shown. [file 40694_2021_126_MOESM5_ESM.jpg]
